# Understanding the impact of the PM4M intervention on women’s mental wellbeing in Zambia and lessons for implementation: a qualitative study

**DOI:** 10.3389/fpsyt.2026.1686644

**Published:** 2026-05-18

**Authors:** Daniela de Vernisy-Romero, Maria Melero-Dominguez, Andrea Fernandez-Rodriguez, Chungu Musonda, Thandiwe Tembo, Mpela Chembe, Mariia Kuleba, Paola del Cueto, Günther Fink, Irene Falgas-Bague

**Affiliations:** 1 Department of Epidemiology and Public Health,Swiss Tropical and Public Health Institute, Allschwil, Switzerland; 2 Department of Psychology, University of Maryland, Baltimore County (UMBC), Baltimore, MD, United States; 3 Department Child and Adolescent Health, Institute of Social and Preventive Medicine, University of Bern, Bern, Switzerland; 4 Graduate School of Health Science, University of Bern, Bern, Switzerland; 5 Chainama Hills College Hospital, Lusaka, Zambia; 6 Zambia-country office, Innovations for Poverty Action, Lusaka, Zambia; 7 Department Epidemiology and Public Health, Household Economics and Health Systems Research Unit, Swiss Tropical and Public Health Institute, Allschwil, Switzerland; 8 Division of General Internal Medicine, Department of Medicine, Massachusetts General Hospital, Boston, MA, United States; 9 Department of Medicine, Harvard Medical School, Boston, MA, United States; 10 Department of Medicine, University of Basel, Basel, Switzerland; 11 Department Research, Swiss Tropical and Public Health Institute, Urban Public Health Group, Allschwil, Switzerland

**Keywords:** common mental disorders, low-middle income setting, maternal distress, psychosocial interventions, women’s health

## Abstract

**Introduction:**

In low-middle income countries (LMICs), Common Mental Disorders (CMD) limit women's ability to care for themselves and their children. Despite high rates of psychological distress among mothers, Zambia’s healthcare system currently can offer only limited support. This study aimed to understand the impact of the Problem Management Plus for Moms (PM4M) psychosocial intervention on mothers of young children and also explored strategies for systemic implementation.

**Methods:**

This qualitative study is nested within a larger clinical trial and aims to qualitatively assess the impact, and barriers for implementation of the PM4M intervention through feedback from 53 women caring for children under 2 years old who received the intervention from Wellbeing community health workers (WCHWs) in community settings in Lusaka from May to August 2023.

**Results:**

Beyond improving mental health symptoms, PM4M was credited with expanding support networks, enhancing financial wellbeing, and fostering empowerment among participants. WCHWs noted personal growth resulting from training and intervention delivery. Both WCHWs and participants stressed the importance of knowledge sharing and involving romantic partners for maximum effectiveness. Identified concerns included the need for neutral spaces during intervention delivery and addressing suicidal behaviors in women and the community. While highlighting the potential of PM4M to boost economic independence and coping skills, the study recognized obstacles to scaling up the intervention. Challenges included raising mental health awareness, combating stigma, and further adapting the program culturally.

**Discussion:**

The study underscores the program's potential impact on CMD in Zambian mothers and calls for sustained efforts in program dissemination and cultural adaptation.

## Introduction

Common Mental Disorders (CMD) constitute a major concern and cause of disability worldwide that disproportionately affects women (1, 2). Risk of CMD among women in charge of young children in sub-Saharan Africa has been significantly highlighted before the COVID-19 pandemic, and even more so after (3). In Zambia, the pandemic significantly worsened the already high distress levels among women caring for young children by causing, for instance, job losses and reduced access to healthcare. Recent findings show that up to 26.1% of women in charge of young children experienced mental health distress in Zambia, with the younger and less educated women facing particularly high risks at par (4). Despite this being an alarming situation, CMD often go unnoticed and largely untreated in the country (5).

The impact of undetected and untreated mental problems in women and their children manifests in multiple forms, reducing wellbeing, food security, and quality of relationships. Mother’s mental disorders may hinder children’s development, heightening their risk of anxiety, stunted physical growth, cognitive impairment, and non-adherence to HIV treatments for both mother and child (6). Evidence on mother’s mental health and their impact on children’s development in Zambia remains limited, but available studies suggest a substantial burden of distress during pregnancy, postpartum, and early motherhood, with prevalence estimates around one quarter to one third in studied samples (4, 7) and strong links to social adversity, low education, high levels of internalized gender stigma, and intimate partner violence (8, 9).

Addressing CMD within its primary care system poses several challenges to the current Zambian healthcare system. As in other LMICs, mental health services are limited, mostly available in specialized institutions and high-level hospitals (10). This lack of integration with the primary care system is due to several reasons, one being that mental health services receive less than 1% of the country’s total health budget. Primary care providers also lack basic training in mental health (11). This leads to a shortage of accessible mental health care, particularly for milder conditions (7). Evidence suggests that culturally adapted psychosocial programs administered by trained non-specialists in communities can alleviate this burden (12, 13). For instance, the World Health Organization’s (WHO) Problem Management Plus intervention (PM+) and the Thinking Healthy program (14) has proven effective and cost-efficient in high-income and various LMICs. However, the need for tailored interventions that address the specific challenges faced by women caring for young children (under 5 years old) in LMIC settings persists (15).

Building on these insights, we developed a program based on PM+ and the Thinking Healthy program delivered by wellbeing community health workers (WCHWs),that aimed to specifically improve the wellbeing of women in charge of young children(mothers) in Zambia. The program addressed key obstacles to intervention engagement for this population, such as domestic responsibilities and childcare demands, while incorporating topics that were specifically of their interest, such as self-care, positive parenting and empowerment strategies The resulting intervention, the Problem Management Plus for Moms (PM4M), was tested in a randomized clinical trial, and demonstrated high feasibility and strong engagement with the intervention, with 80% of participants completing at least six sessions and reporting high satisfaction levels. Moreover, the intervention showed promising outcomes, with significant reduction of mental distress in treated women when compared with the control group (16, 17). Prior to the intervention participants faced mental health challenges related to depression and anxiety, and experienced physical symptoms related to mental health distress, such as poor sleep quality and a diminished appetite, as confirmed by their SRQ-20 scores being greater than 7. The findings of this intervention showed a significant decrease in SRQ-20 scores of the intervention group compared to the control group, with the adjusted OR of high mental distress being 0.5, proving the program’s ability to help treat symptoms related to mental health distress However, the intervention’s full impact, and its potential implementation future cannot be determined without a better understanding of women’s perception and feedback from the intervention providers (WCHWs).

The aim of this study is to qualitatively assess the feasibility, acceptability, impact, and scalability of PM4M intervention on mother’s wellbeing in a representative sample of women living in urban Zambia. The research team acknowledges that some primary authors are trained within Western academic traditions, which may shape initial interpretative lenses. To address this and strengthen cultural validity, the analytic process was intentionally collaborative and iterative. The broader author group reflects diverse cultural and professional backgrounds, including three Zambia-based co-authors with deep contextual knowledge. These co-authors actively reviewed and provided feedback on the codebook, thematic structure, and interpretation of findings. In addition, the senior author’s extensive experience working in the Zambian context further informed the analytic process. This reflexive and collaborative approach helped mitigate potential bias and enhanced the cultural relevance and credibility of the analysis.

## Materials and methods

### Study design

This is a qualitative study nested in a larger clinical trial aiming to develop and assess a culturally adapted intervention designed to alleviate distress in mothers of children under the age of 2 (NCT05627206, registration date 2022-11-07). This qualitative research complemented outcomes on the feasibility (barriers to participation), acceptability (engagement), impact (perceived benefits, mechanisms of impact) and scalability of the intervention (implementation challenges, and potential refinements) through open-ended questions included in the participants’ follow-up interviews and a focus group with the WCHWs who delivered the intervention.

### Participant recruitment, intervention and procedures

A total of 265 mothers with mental health needs (defined as SRQ-20≥7) reported in the parent trial were randomly assigned with equal probability to the intervention (N = 134) or control group (N = 131). As described in further detail in previous work (17) the intervention group received a locally adapted version of the PM+ intervention, the PM4M, specifically designed for women with young children living in Southern Africa. It combines elements from the PM+ intervention and “Thinking Healthy” intervention developed by WHO (18, 19) with some selected components of the Strong Minds-Strong Communities psychosocial intervention (20). To develop the PM4FM intervention following the Castro-Barrera framework for cultural adaptation (21). First, we reviewed existing literature on psychosocial interventions in low-income settings, cultural expressions of common mental disorders among women in Southern Africa, and key sociocultural stressors, and complemented it with focus groups and interviews with Zambian mothers with lived experiences. Second, we integrated core PM+ components with elements from the *Thinking Healthy* manual (e.g. psychoeducation on child development and importance of mother-child interaction) and Strong Minds-Strong Communities intervention (e.g. psychoeducation about healthy habits), adapting language and adding practical guidance to support delivery by WCHWs. Third, we incorporated feedback from the research team, local experts, and WCHWs, including the addition of culturally relevant case vignettes to enhance understanding and applicability. Finally, we pilot tested the manual with women with mild symptoms and refined it based on their feedback. The resulting intervention combines psychoeducation, problem-solving, cognitive restructuring, motivational interviewing and mindfulness exercises to address maternal distress and improve coping strategies. It follows a collaborative and patient-centered approach where an individual’s needs are central, and contents of the intervention are adapted to each person.

In comparison with the original PM+ that consists on five individual sessions lasting one hour and a half, the PM4M intervention is provided mostly individually (sessions 1 and 10 can be delivered in groups of a maximum of 8 women, but sessions 2 to 9 are delivered individually). It is organized in ten 1-hour sessions, and provided on a weekly basis by a specifically trained and supervised WCHW in women’s homes and/or by phone. Group sessions are provided in community healthcare centers close to their residence.

Five WCHWs were trained over four days, followed by supervised role-playing and weekly supervision to ensure fidelity. They were selected from a pool of candidates with at least a high school diploma, fluency in English and Nyanja, and prior community experience, based on interest, availability, and performance in a basic skills assessment. The training combined instruction on program objectives, ethics, emergency procedures, and core psychotherapy skills (e.g., motivational interviewing, problem-solving), with applied practice on session content and managing complex situations (e.g., domestic violence, suicidality). This was followed by four weeks of structured role-plays with weekly group supervision, conducted by local clinicians and the research team, with continued supervision and fidelity monitoring throughout implementation.

Of the 131 women assigned to the intervention group, 73 were excluded because of not being located, due to ongoing mental health treatments, severe disorders, or substance misuse. The rest, 61, were offered the intervention and all of them received at least one session. Figure 1 of the parent trial shows the participant flow of the trial in detail (17). Only the 61 participants who were offered the intervention were approached during the 6-month follow-up assessment, as part of the post-intervention assessment, to provide qualitative feedback. Trained research assistants conducted open-ended interviews in Bemba, Nyanja, or Tonga, depending on each participant’s language preference. A total of 53 participants consented to respond to the qualitative questions. Their responses were audio recorded and later transcribed into English by the same team of bilingual research assistants. More details on the development, specific content, training, and supervision of the intervention can be found in a previous publication (16, 17).

### Measures

Feedback on the PM4M intervention was gathered through both structured survey forms and focus groups. The survey included open-ended questions that explored participant’s opinions about thefeasibility, acceptability, and potential barriers to scaling up the intervention. Feasibility was assessed by querying on difficulties in completing the initial session and identifying the most beneficial aspects of session 1. Acceptability questions focused on engagement with the program (e.g. “If you were not able to complete at least 6 of the 10 intervention sessions, why do you think this happened?”). The form also included questions about the perceived impact of the intervention on participant’s wellbeing and potential mechanisms for that impact (e.g. “Is there something you have done or a change you have made in your life as a result of the intervention?”). Finally, the participant was asked about scalability of the intervention. The full list of the open-ended questions can be found in the **Supplementary Materials**.

To complement participants’ survey-based feedback, the research team conducted a focus group with the WCHWs who delivered the intervention (N = 5). The focus group was conducted in two parts using a semi-structured interview, led by trained research staff (MM-D, DdV-R, and PdC). The focus group’s semi-structured guide was developed in English. The guide was organized around three main domains: feasibility (e,g., barriers to delivering sessions), acceptability (e.g., cultural appropriateness and helpfulness of materials), and satisfaction with training and supervision. Questions such as, “If you could change any aspects of the intervention, what would they be?” were used to prompt discussion across these areas. A copy of the semi-structured interview can be found in the **Supplementary Materials**.

### Analysis

We used a qualitative thematic analysis that followed established procedures (22) (14). Following an in-depth analysis of the transcriptions, a codebook was developed by the main authors of the study. Coding followed a deductive approach where initial codes were informed by previous literature and the study objectives. The codes were then refined through repeated engagement with the data and collaborative review. This codebook was continually refined by the research team during their weekly meetings as coding and analysis progressed. Data saturation was reached when no new concepts or themes emerged across transcripts, both within participant and WCHW groups separately, as well as combined. Two trained research team members (DdV-R, MM-D) independently coded the transcribed data. Coding was conducted within shared digital environments that supported collaborative review, coding, and iterative refinement of emerging themes. This process was later reviewed by a third team member (IFB), who carefully examined the coded data, resolved any discrepancies and validated the findings. The research team further reviewed and refined the themes to formulate the findings detailed in this article.

## Results

**Table 1** maps the major themes, their subthemes and representative quotes that frame the structure of the results.

**Table 1:** Summary of major themes, subthemes and illustrative quotes from the qualitative analysis of the PM4M intervention’s impact on women’s mental wellbeing in Zambia.

Theme 1: Factors Driving Positive Outcomes of the PM4M Intervention
**WCHW-participant relationship** “*They encouraged me after I shared my problems, which helped me to stop thinking about ending my life … the WCHW’s call gave me hope as well.” (Participant 32)* **Use of core intervention techniques** *“They are able to distinguish between which thoughts are helping them and which thoughts are not helping them.” (WCHW 4)*

Theme 2: Understanding the Positive Impacts of PM4M
**Improvement in mother-child relationships** *“There's a great change in how I used to treat my children. I used to beat them, but now I know how to control myself.” (Participant 1)* **Positive mental and physical health improvements** *“I changed how to handle my thoughts and emotions and how to keep going, keep doing. Never to give up no matter the situation I find myself in.” (Participant 2)* **Improvement in social support and relationships** *“There is a great change. I did not know how to relate well with my neighbors, but after learning about social support, I now know how to relate and talk to them, chat with them, and seek help from them.” (Participant 5)* **Positive financial impact through increased empowerment.** *“The way I approach my business is now different." (Participant 37)* **Positive impact on WCHWs’ wellbeing** *“It has really worked for me.” (WCHW 4)*

Theme 3: Intervention Delivery and Format. Key Points for Engagement Success
**Format of the intervention and materials** *“[Group sessions] helped to push the objectives of the program because people were able to open up as they realized they were not the only ones with this problem.” (WCHW 2)* **Supervision aspects and training** *“Whether I would have trouble or where the client was not responding and I would feel like giving up … the supervisor would guide me through it, and he/she would check up and see.” (WCHW 3)* **Changes related to supervision and training** *“You would even have to reschedule your sessions because you don’t have the necessary resource at that particular time.” (WCHW 2)* **Changes related to session’s content** *“[ … ] Some of them [the participants] could not relate with some activities [ … ]” (WCHW 3)*

Theme 4. Recommendations for Scaling PM4M
**Supporting financial independence and empowerment** *“Because staying without doing anything is what brings about most bad thoughts.” (Participant 49)* **Expanding program reach** *“To continue and also make us teachers in the community.”* (Participant 21) **Addressing intimate relationship problems and incorporating partners in treatment** *“Include our partners in the program. Even through the phone. Include men because even the funeral at the next door is a male suicide, they are also much stressed.” (Participant 31)*

WCHW responses did not require translation, as WCHWs answered the questions directly in English.

Participant responses were originally in Bemba, Nyanja, or Tonga and were then translated into English by bilingual research assistants.

Bold headings summarize participants' key perceptions.

### Theme 1: Factors driving positive outcomes of the PM4M intervention

#### Relationship between WCHWs and participants

When asked about the reasons why they thought PM4M made a positive impact, women reported that enhanced mental health outcomes were related to the low-barrier approach the WCHWs followed to keep them engaged in the program. The majority of participants highlighted the importance of their good relationship with their WCHWs as a driver of their improvement. They reported that the WCHWs helped them modify their way of thinking, encouraged them to challenge problems they were facing in their lives, and made them look forward to attending the sessions. Participants commended the effective communication, organizational skills, and adept teaching abilities of the WCHWs, noting their patience, persistence, kindness, dedication, and empathetic understanding throughout the intervention.

“They encouraged me after I shared my problems, which helped me to stop thinking about ending my life … the WCHW’s call gave me hope as well.” (Participant 32).

“It has changed, I used to be moody, and run away from the WCHW, but after she persisted, I have learnt how to break my moody self and started enjoying the program” (Participant 10).

##### Use of core intervention techniques

WCHWs noticed a relationship between the great improvement in their participant’s wellbeing and the participants’ implementation of the intervention techniques and exercises. They emphasized the psychoeducation components explaining psychological wellbeing pathways, like the cognitive restructuring model and behavioral activation.

“The CBT Triangle. You see that actually, participants become aware that the way they feel, the way they think and their behavior, it is something that is interconnected.” (WCHW 2).

WCHWs also mentioned that the intervention components helped participants increase control over their lives through regulating their thoughts and emotions.

“…they are able to process their thoughts well, they are able to distinguish between which thoughts are helping them and which thoughts are not helping them.” (WCHW 4).

Techniques like breathing exercises, stress management using the “stop light” method, problem-solving strategies, and relatable narratives such as “Mary’s story” were highly praised. Components including managing circles of control, addressing negative thoughts, problem-solving methods, behavioral activation, promoting children’s positive reinforcement, and nurturing social support garnered widespread recognition among participants.

“My thoughts have changed, I was encouraged. The way I am supposed to behave when I am upset has changed” (Participant 40).

“When I am stressed I used to do the breathing exercise and I would have a calm mind and think positively afterwards.” (Participant 33).

WCHWs highlighted an improvement in their participant’s marital relationships, specifically in learning to process and understand the intent behind their partner’s actions instead of jumping to quick, irrational conclusions.

“…when we did the session, [ … ], she was like, I really really appreciate this because I was so aggressive when I fought with my husband, I would beat up the kids, but now I can breathe, I can relax.” (WCHW 3).

### Theme 2: Understanding the positive impacts of PM4M

Through this in-depth analysis of participant and WCHW responses, we can understand the nuances of the PM4M intervention’s impact beyond the improvement in mental health outcomes.

#### Improvement in mother-child relationships

Participants reported that throughout the program they learned helpful strategies to take better care of and spend more quality time with their children.

“I managed to concentrate towards my children after I was taught about Mary’s story.” (Participant 39).

Furthermore, they reported that through the intervention, they had gained skills in managing their own emotions in the presence of their children and enhancing their overall interactions through more assertive and calmer communication. Several participants highlighted the positive rewarding technique and the role model example through Mary’s case as the most helpful techniques from the manual in improving their relationship with their child. WCHWs also noticed an improvement in the participant’s relationships with their children, specifically in their abilities to constructively influence their children’s behaviors and subject them to fewer physical punishments.

“There’s a great change in how I used to treat my children. I used to beat them, but now I know how to control myself.” (Participant 1).

#### Positive mental and physical health improvements

The intervention improved both the mental and physical health of the participants. Women reported that the intervention helped them divert suicidal thoughts, identify and redirect low mood, stay calm under pressure, successfully problem solve, and allow negative opinions from others to have less influence over their emotions.

“I changed how to handle my thoughts and emotions and how to keep going, keep doing. Never to give up no matter the situation I find myself in.” (Participant 2).

Aside from their mental health, participants additionally noted a positive change in their physical health, citing improvements in sleep, heart health, body weight, blood pressure, and exercise habits.

“I used to have heart issues but now I don’t stress. I improved health wise, even gained weight” (Participant 29).

#### Improvement in social support and relationships

Both participants and WCHWs observed intervention-related improvements in their social support networks and intimate relationships. Participants found that after the intervention they were able to communicate and relate better with family, gain more support from their husbands and overall improve their marital relationships. Participants reported increased ability to keep friends and share problems with them, get along with neighbors, seek help from and lean on others.

“There is a great change. I did not know how to relate well with my neighbors, but after learning about social support, I now know how to relate and talk to them, chat with them, and seek help from them.” (Participant 5).

#### Positive financial impact through increased empowerment

When asked about indirect benefits from the intervention, participants reported notable financial improvement. They reported that since participating in the PM4M intervention, they began growing vegetables for household consumption and for sale. Others transitioned to jobs with better financial prospects. Two participants mentioned that the intervention taught them how to not give up when facing challenges in their businesses, enabling them to sustain their entrepreneurial efforts and achieve longer-lasting profits than before. Participants reported that, through the PM4M intervention, they gained a sense of autonomy and self-confidence, empowering them to start and maintain businesses while improving their ability to save money.

“I have stopped the job I was doing and have started a business, which is sustaining me rather than that stressful job where I was not getting paid a lot of money. I can now save money, and I keep on going.” (Participant 34).

“The way I approach my business is now different” (Participant 37).

#### Positive impact on WCHWs’ wellbeing

The program positively impacted not only the participating women, but also the WCHWs providing the sessions. They applied strategies from the training, the manual and sessions to their own lives, and to those of their friends and family. One WCHW shared how the program inspired her to prioritize her wellbeing, motivating her to download and regularly use a relaxation application.

“What I learned about the training was empathy. And the intervention as a whole. I learned how to be empathetic and not sympathetic.” (WCHW 5).

“It has really worked for me … I did not know about the circles of control but I learnt and I feel more positive … I have noticed a shift in perspective towards people on how I can interact with them… (and) also my social support … my social network has increased.” (WCHW 4).

### Theme 3: Intervention delivery and format. Key points for engagement success

Participants and WCHWs valued the intervention materials and hybrid format, though group session management and attendance posed challenges. While phone sessions improved accessibility, in-person meetings enhanced engagement but raised confidentiality concerns. Supervision and training were essential, with calls for more consistent support. Lastly, cultural adaptations are needed to align some health recommendations with participants’ realities, ensuring relevance and feasibility.

#### Format of the intervention and materials

Both participants and WCHWs reported that the intervention package was engaging, and useful. They positively valued the manual writing style, pictures and formatting, and accompanying materials (participant workbook and fidelity forms) for their guidance and pivotal role in guiding individuals back on track whenever the conversation veered off course, especially when the intervention session was provided over the phone. While the fidelity form proved extremely useful for pacing the delivery of the intervention and signaling specific aspects that needed improvement, opinions diverged on the program delivery format. Although the WCHWs particularly valued the hybrid approach, which started with a group session and transitioned to individual ones, some faced challenges managing and delivering group sessions, especially groups with low participant attendance. They reported difficulties prompting participants to speak more, share their experiences and manage session duration. Conversely, other WCHWs advocated for more group sessions, citing their ability to reduce isolation and foster mutual understanding among participating women.

“[Group sessions] helped to push the objectives of the program because people were able to open up as they realized they were not the only ones with this problem”. (WCHW 2).

This sentiment also resonated with participants, who highlighted the value of connecting with other women with problems like theirs, leading to a sense of empowerment.

“I learnt that I can control myself even when I’m passing through a problem because I heard other people’s problems” (Participant 34).

The choice between in-person or phone sessions also sparked debate among the WCHWs. While some favored the phone option for its convenience in reaching participants who lived in remote locations, others emphasized the benefits of in-person meetings. The latter was particularly valued for participants without phone access or for whom face-to-face interaction facilitated better connection and engagement, as one of the WCHWs mentioned.

“But for the physical session, you will see that the participant is actually fully attentive and you’re moving together, they’re able to understand.” (WCHW 2).

Overall, participants unanimously viewed the program as effective and not needing large modifications. However, WCHWs encountered challenges in establishing consistent communication channels with participants. Frequent changes in residence without notice or the absence of a fixed address were common and affected retention to treatment. One proposed solution involved offering phones to participants to facilitate direct communication and reduce their reliance on personal phones. On the other hand, a concern raised by the WCHWs pertained to confidentiality during in-person sessions at participants’ houses as sometimes they were disrupted by spouses or neighbors. To mitigate this, they advocated conducting sessions in neutral spaces.

“Most of the time [ … ] we were doing the sessions in their homes [ … ] so you’ll find that the husband is around and [ … ] it compromises [the session]”. (WCHW 2).

“I was away [outside the house] most of the time, because the pressures I had, and I was mostly in a noisy place when the WCHW was calling.” (Participant 42).

#### Supervision aspects and training

All WCHWs agreed that the supervision meetings were valuable, offering clear guidance and support in handling difficult situations.

“Whether I would have trouble or where the client was not responding and I would feel like giving up … the supervisor would guide me through it, and he/she would check up and see.” (WCHW 3).

Regarding the training, the WCHWs found it beneficial and practical, although intense. They emphasized its practicality, going beyond simply reading the manual. WCHWs asserted that without the training, delivering the intervention effectively would have been impossible to achieve.

“Imagine if we just got the manual and started talking to the people, it would have been all over the place. So, the role-play and the training that we did, it really helped, especially with the screenings that they made us go through. So, we’d prepare ourselves and just practice before going” (WCHW 3).

#### Changes related to supervision and training

WCHWs did not express the need for any significant changes regarding supervision and training. However, most agreed that supervision should be consistent and ensured through providing resources (phone data), and that issues should be addressed more quickly.

“It’s something that should have been a consistent kind of flow because we were constantly using talk time whether we were doing physical visits, whether we were doing phone sessions, so we would request for maybe talk time, but it would take some time to come to probably you would even have to reschedule your sessions because you don’t have the necessary resource at that particular time.” (WCHW 2).

#### Changes related to session content

The WCHWs emphasized the need for further cultural adaptation of the intervention content on healthy habits to better align with the local context and some women’s clinical severity. For instance, nutritional content lacked awareness of challenges for targeted women in accessing fruits or protein sources, and proposed activities such as jogging or going for a drive were not relatable to most women in their context.

“[ … ] Some of them [the participants] could not relate with some activities [ … ] I think they’ll think you’re pushing them too far. They’re telling you that they’ve slept hungry and you’re telling them to go jogging. So, I think some of those things were not culturally adapted. The things that they would love like gardening were okay. Things like gardening or just leaving the house to take a walk and reflect, just get a different feel in different environments. And also the fruits mentioning they can’t make sure you have fruits every day so that they eat healthy. [ … ] y “(WCHW 3).

### Theme 4: Recommendations for scaling PM4M

Participants and WCHWs provided several recommendations related to financial empowerment, expanding the program’s reach, and involving partners and household members in the intervention to improve and scale up the PM4M intervention.

#### Supporting financial independence and empowerment

Both participants and WCHWs unanimously emphasized the link between financial empowerment and employment and improved mental wellbeing, underscoring the need for the integration of such social determinants into the intervention. In essence, they emphasize the vital connection between having an independent financial purpose, an occupation and improving mental health. As a participant reported:

“Because staying without doing anything is what brings about most bad thoughts” (Participant 49).

“Empowering Women with capital, entrepreneurship skills and funding for them what can keep them busy so that they are able to take care of their children and not go into alcohol abuse” (Participant 34).

#### Expanding program reach

A majority of the participants expressed that the PM4M program should be scaled up. Some participants further suggested that former participants from the program could be trained as peer WCHWs so they could share what they have learned with others.

“To continue and also make us teachers in the community” (Participant 21).

“Recruit us to teach others because a lot are committing suicide, it involves a lot of people” (Participant 25).

This was also noted by WCHWs, who expressed the urge to share what they have learnt, act as role models and empower the community as a whole.

“As Zambians when you are empowered with something, it’s something that they tend to put to work together and wanting to tell others to say please, this kind of life is really healthy and it’s really helping. Seeing our families that they come together, our children, our spouses, that love and that healthy kind of living. (…), I’m very sure they’ll do greater things more than we even have done to them because they’ll go out there to teach the same things that we were taught.” (WCHW 1).

Mental health literacy and stigma presented significant challenges for reach and treatment. WCHWs recognized the importance of early treatment and recommended improving the referral processes through working towards reducing stigma, as many women were hesitant to accept referrals to psychiatric clinics.

“A lot of people are not used to having counseling or receiving any kind of therapeutic intervention in terms of mental health, because for them, they understand that counseling is mostly attached to HIV and AIDS.” (WCHW 2).

“They have the perception that when someone is going to mental health facilities, maybe they have diagnosed them to be mad or insane” (WCHW 4).

#### Addressing intimate relationship problems and incorporating partners in treatment

There is a consensus with the WCHWs that intimate partners and the people sharing a household with the mothers should participate in the program. Cohabitation issues arise as a frequent source of conflict that affects participants’ wellbeing and children’s development. Thus, most participants and WCHWs agreed on the need to expand the PM4M to partners and members of the same household:

“I think I would add in future, I would add that person [living with the participant] just for them to know how they’re going to work together and try to live in a more decent environment. Because it seemed like we were trying to work one person when everyone around them was like falling apart and creating commotion” (WCHW 3).

“So if the mother is doing that and the husband is doing the opposite, I think the child might get confused but the two of them may be talked to and guided also, if there was a male intervention they could work together for the wellbeing of the child.” (WCHW 3).

“Include our partners in the program. Even through the phone. Include men because even the funeral at the next door is a male suicide, they are also much stressed.” (Participant 31).

## Discussion

This study analyzed the participant and WCHWs’ perspectives of the PM4M intervention. The feedback received indicates improvements in mother-child relationships, physical and mental health, social support and relationships, and financial empowerment. The intervention was able to obtain positive outcomes through its psychoeducational content, techniques and exercises and through the strong relationship fostered between participants and WCHWs, whose teaching and organizational skills were emphasized as crucial for the program’s success. Suggestions for improvement include changes to the intervention delivery format and location, increased cultural adaptation and inviting partners, and increased reach to scale up the intervention.

The program’s techniques, like cognitive restructuring and breathing exercises, encouraged participants to apply these methods to real-life situations. This client-centered approach helped the participants gain control over various aspects of their lives by improving their ability to manage emotions and thoughts (23). As a result, the benefits extended to their intimate relationships, significantly impacting their overall wellbeing. These results align with previous studies, showing that non-specialist providers can effectively deliver mental health interventions, offering a promising solution to the mental health gap in lower-middle-income settings (24).

The intervention improved participants’ ability to take care of and spend more time with their children. Examples include enhanced regulation of emotions when interacting with their children, more use of effective communication, and fewer physical punishments. Similar results were observed when providing the original PM+ intervention to a population of migrant and refugee women in northern Colombia where women demonstrated a higher patience level, stronger communication, and fewer physical punishments towards their children as result of the intervention (25).

Similar to previous studies testing the PM+ intervention, participants reported improved mental and physical symptoms with diversions of suicidal thoughts, a greater control over their emotions and mood, improved body weight, and better sleep (26). This supports the evidence on using adapted versions of PM+ to improve both the mental and physical health of low-resourced populations with limited access to mental health professionals. For Zambians, community and social support play an important role in everyday life (27). Friends, family, and entire neighborhoods and communities often rely heavily on each other, a sentiment emphasized by both participants and WCHWs in this study. In fact, both groups noticed an improvement in their own social support and relationships as a result of the intervention, citing improvements in their relationships with their friends, families, marital partners, and neighbors. However, WCHWs did point out that participant stigma towards mental health did make it more challenging to reach them and facilitate treatment, a sentiment echoed in a study in Nakuru County, Kenya, where stigma and societal expectations for men to be stoic significantly reduced their engagement with PM+ programs aimed at supporting survivors of gender-based violence (28).

Participants reported increased confidence in financial decision-making and business pursuits as a result of the intervention. While positive, this finding was unexpected since no session specifically addressed financial empowerment. Although not initially expected, this theme aligns with frameworks on the social determinants of mental health, which identify economic stability and employment as central influences on psychological wellbeing (29). The financial gains and increased autonomy reported by participants reflect improvements in these determinants and help explain the broader mental health benefits observed. Given its strong link to overall wellbeing and the well-established bidirectional relationship between poverty and common mental symptoms (30, 31), this outcome suggests the PM4M intervention may also positively influence financial wellbeing among women with children in low-income settings—an area still underexplored in LMICs. Emerging evidence indicates that psychological interventions can enhance agency, self-efficacy, and future-oriented thinking, which may indirectly support economic behaviors (32). Future research should quantitatively assess this impact and explore adaptations that incorporate economic literacy or livelihood components. From an implementation perspective, integrating PM4M within existing social protection or financial support programs could be particularly impactful, as prior studies in LMICs show that combining psychological and economic interventions can produce synergistic improvements in both mental health and economic outcomes (33, 34).

Overall, the intervention was praised for its comprehensive material, including the manual, participant workbook, and fidelity forms, which were expected to yield solid results based on the growing literature from the programs they are based on (14). The low-barrier, flexible delivery approach combining group and individual sessions, along with the hybrid delivery format (remote or in-person), was perceived as beneficial, though challenges were identified. Even though there were identified benefits of the group sessions, managing them was difficult for some of the WCHWs. Previous studies have pointed out that an important factor contributing to WCHWs’ competency was the simplicity of the intervention (35, 36). Group sessions may add an extra layer of complexity that requires specific training in group management to overcome the challenges inherent to this form of intervention. Thus, specific training on group sessions provision should be included in the future PM4M training curricula.

Although previous research on the ability to adapt PM+ to be delivered through the phone in coastal Kenya (26, 37) highlights the advantages of phone-based interventions—such as flexible scheduling, accessibility from preferred locations, and ease of use—our study identifies key disadvantages specific to impoverished settings. Addressing these challenges will be crucial for improving future implementation efforts. In Lusaka’s low-income compounds similar to other urban and suburban areas of Southern Africa, many women do not have their own phone, failing to reserve time for interventions, and lack a private space to avoid being overheard by others, which significantly impacts confidentiality.

Supervision and training processes were generally seen as positive and effective. Despite this, there was a call for more consistent and timely supervision. Providing effective supervision to lay workers delivering psychosocial interventions is a well-documented challenge (38). Key barriers include the loss of non-verbal communication in phone-based meetings and supervisors’ competing responsibilities due to the shortage of mental health professionals (39). These issues are particularly pressing in LMICs, where clinicians qualified for supervision are often overworked and scarce. Beyond increasing resources, these challenges could be addressed by enhancing WCHWs’ capacity through training in basic suicide management skills (40), utilizing telemedicine for supervision (30, 41), or adopting a train-the-trainer approach, where the most capable WCHWs are trained to become future supervisors (42). Further research is needed to evaluate the feasibility and effectiveness of these strategies in low-income settings.

### Considerations for scaling PM4M intervention

WCHWs and participants’ responses encouraged scaling up the intervention, advocating for first expanding the program to include intimate partners. Involving intimate partners could significantly enhance program outcomes, as seen in successful applications within antenatal care (43) and HIV prevention programs (44). However, future research should investigate how this involvement of partners can be achieved while keeping women’s privacy and empowerment outcomes. Both participants and WCHWs proposed training former recipients of the program as WCHWs for expanding the reach and implementation. This would be a feasible solution following the extensive literature on peer-delivered psychosocial interventions in LMICs (45). Integrating the intervention into primary care, or into specific and already well-established healthcare programs such as maternal health, or early childhood development services appears to be a natural and feasible pathway to achieving the increased reach requested by participants and WCHWs. Further recommendations included increasing content and training for suicide prevention because of the high occurrence of suicide ideation within the Zambian community.

This study has shown promising results in providing a scalable, culturally adapted intervention aimed at improving the quality of life for mothers in Zambian settings. Delivered by paraprofessionals, this approach is a promising strategy to close the mental health gap in lower- and middle-income settings. It has not only enhanced the mothers’ mental wellbeing but also positively impacted on their marriages, social support, and relationships with their children. Additionally, participants emphasized the need for similar programs and expressed a desire to make them more accessible to others in their community, demonstrating high acceptance of the program. To our knowledge, this study is one of the first to explore the benefits for both participants and professionals, as well as the drivers behind these benefits.

However, it is important to note the study’s limitations, first, the lack of in-depth interviews with individual participants that would have provided more detailed information. Although we used open-ended survey questions in our follow-up interviews that gathered crucial feedback on a variety of important topics, in-depth interviews with each participant would have allowed for deeper probing into participant responses and emotions, greater clarifying the gathered results and reducing ambiguity. The level of probing in-depth interviews allow for could have also reduced potential social desirability effects, especially for more sensitive topics. Additionally, in-depth interviews with individual WCHWs could have increased engagement and response rates, as sometimes individuals are inclined to speak less when in focus groups with others. Second, even though we contrasted our results with the Zambian team members, the coders of the qualitative data are originally from western countries and do not share the cultural background of participants and WCHWs, which can be a crucial factor that influences the appropriate analysis of findings. Though coders were informed of and sensitive to Zambian culture and belonged to a diverse research group, the research team acknowledges this could have affected the interpretation of participant and WCHW responses as well as the findings, potentially leading to translation bias. Furthermore, focus groups were conducted in English, which is the second most spoken language in Zambia. While WCHWs were fully fluent in English, conducting the focus groups in Bemba, Nyanja, or Tonga could have facilitated the expression of their emotions and opinions.

## Conclusions

This study demonstrates the potential of the PM4M intervention to address mental health needs for mothers in Zambian communities and is perceived very positively by the treated women and the WCHWs. The program successfully enhanced participants’ mental and physical health, relationships, parenting practices, and even financial empowerment. The intervention’s culturally tailored, patient-centered approach, combined with the strong rapport fostered between participants and WCHWs, underscores its relevance and effectiveness in resource-limited settings.

Recommendations for future efforts include integrating PM4M into primary care systems and addressing community-specific needs. These findings are significant as they highlight scalable, sustainable strategies to bridge the mental health gap in low- and middle-income countries, while emphasizing the importance of community support and patient-centered delivery. Despite limitations, this research provides valuable insights into how mental health interventions can transform lives and strengthen families and communities.

## Data Availability

The raw data supporting the conclusions of this article will be made available by the authors, without undue reservation.
