# Supplementary material for: Understanding the impact of the PM4M intervention on women’s mental wellbeing in Zambia and lessons for implementation: a qualitative study
Source: Front Psychiatry. 2026 May 18;17:1686644. doi: 10.3389/fpsyt.2026.1686644 (PMC13222936; doi:10.3389/fpsyt.2026.1686644)
Supplement: Supplementary file 1 [file SupplementaryFile1.docx]

**Supplementary Materials**

**TITLE:** Understanding the Impact of the PM4M Intervention on Women’s Mental Wellbeing in Zambia and Lessons for Implementation: A Qualitative Study.

**AUTHORS:** Daniela De Vernisy-Romero*, Maria Melero-Dominguez*, Andrea Fernandez-Rodriguez, Chungo Musonda, Thandiwe Tembo, Mpela Chembe, Mariia Kuleba, Paola del Cueto, Gunther Fink, Irene Falgas-Bague

*co-first authors

**Supplemental Materials** for ***“****Understanding the Impact of the PM4M Intervention on Women’s Mental Wellbeing in Zambia and Lessons for Implementation: A Qualitative Study.”*

**Table of Contents**

[1. PMFM WCHW Post-Intervention Focus Group Interview Guide 3](#_Toc218580726)

[2. Participant Post-Intervention Open Questions 6](#_Toc218580727)

[3. Important Quotes by Theme 6](#_Toc218580728)

[Theme 1: Factors Driving Positive Outcomes of the PM4M Intervention 6](#_Toc218580729)

[Theme 2: Understanding the Positive Impacts of PM4M 12](#_Toc218580730)

[Theme 3: Intervention Delivery and Format. Key Points for Engagement Success. 14](#_Toc218580731)

[Theme 4. Recommendations for Scaling PM4M. 20](#_Toc218580732)

# 1. PMFM WCHW Post-Intervention Focus Group Interview Guide

**Before starting recording**

Welcome to our focus group discussion! Before we begin, we would like to obtain your verbal consent to record this session. We will use this recording to be able to refer back to the comments made during today’s discussion, which will help us in the analysis. Although we are recording, today’s session will remain completely confidential. We will not be using any personal identifiable information (such as name, address, etc.) in the analysis or in anything related to the study. Does everybody consent to this focus group Zoom session being recorded?

In a moment we will start the recording and ask everyone individually whether they consent to be recorded during today’s session. We will now call on each of you by your name so that you can let us know whether you consent.

**Start the recording**

Today is () day at () time. This is the PMFM WCHW Post-Intervention Focus Group.

Do you consent for this focus group Zoom session to be recorded?

**Welcome/Topic Introduction**

Thank you for taking the time to be here with us today. The purpose of this session is to get your feedback about your experience being a WCHW in the PMFM intervention. This session will last approximately one hour.

I will first introduce myself. I am Maria del Sagrario and I will be the moderator in today’s session. I am also with Daniela, who will also be a moderator today. We also have with us Paola del Cueto who will help us to facilitate the session, and Mariia who is also a researcher who will be observing today’s session.

The format we will be using today is that of a focus group. This means that we will have a conversation focusing on specific questions related to the PMFM program, which will remain confidential. We would like to create a safe environment, in which everyone feels comfortable answering all questions and having open discussions with each other.

My main role in today’s session will be to ensure that everyone is able to participate and that we work our way through all of the questions.

**Guidelines**

Before we begin, we would like to go over some guidelines for participating in today’s conversation.

- First, there are no right or wrong answers to our questions, only differing points of view. Keep in mind that we are interested in your opinion, whether that be affirmation or critique. Please know that at times the negative comments are the most helpful.
- To ensure everyone can be heard, we ask that only one person speak at a time. Feel free to raise your hand if you want to add something.
- We will be audio recording this session in order to not miss any of your comments. The recording will only be used by study staff.
- We ask that you silence or turn off your phones or devices that have the potential to distract you or the group.

Before we begin, we would like to ask you all to say your names.

**Key Questions**

1. How do you feel the sessions went?
2. Which sessions, if any, did you find to be the most beneficial/interesting for the participants? And the least?
3. What was one obstacle you encountered when delivering the intervention and how did you work around it? – *Note to the facilitator -* make sure to get specific examples
4. If you could change any aspects of the intervention, what would they be?
5. What intervention materials did you find most helpful? And the least?
6. Did you find the intervention to be appropriately culturally adapted to the backgrounds of the participants?
7. The intervention consists of 10 sessions (8 individual and 1 or 2 group). Was this enough, too few, or too many to accomplish the goals of the program?
8. Overall, did you find the length of the sessions to be too short, too long, or about right?
9. Do you have any further comments that could be of use to us when improving the intervention? What do you think is missing? What would you add?
10. How helpful was the WCHW training in preparing you to deliver the intervention to the participants? (Did it prepare you well enough to be able to face the challenges that you came across?)
11. What did you learn from the WCHW training that will help you in the future when assisting patients/participants/people? Is there anything that you can use on yourself?
12. Did you get enough support from your supervisor? Is there anything you would change about the supervision you received?
13. Do you have any further comments that could be of use to us when improving both the WCHW training and the supervision? What do you think is missing? What would you add?
14. Did you encounter any challenges with management or the organization that you worked with?

**Closing Statement**

Thank you all for your participation in our focus group. We appreciate each of your contributions and look forward to using them to strengthen the program. Please feel free to reach out if any questions or comments arise. Have a great rest of your day!

**This is the end of the recording.**

# 2. Participant Post-Intervention Open Questions

**Feasibility:**

- Did you have any difficulties in completing the first session of the intervention? For example, transportation, access to a mobile phone, little time, etc.
- What did you find most helpful about the first session? For example, communication with the WCHW, being able to identify with the intervention and the examples etc.

**Acceptability:**

- If you were not able to complete at least 6 of the 10 intervention sessions, why do you think this happened? If you were able to complete at least 6 of the 10 intervention sessions, what helped you? For example, your relationship with the WCHWs, your feelings towards the program, etc.

**Satisfaction:**

- From 1 to 5, with 1 being the least and 5 being the most, how satisfied are you with the intervention?
- From 1 to 5, with 1 being the least and 5 being the most, how satisfied are you with the ability of your WCHW to help you through the intervention?

**Overall Feedback:**

- How do you think this intervention has affected your relationship with your child? For example, do you think it has improved, stayed the same, or changed in any way? Can you give us an example?
- How do you think this intervention has affected your wellbeing? Could you give me an example?
- Is there something you have done or a change you have made in your life as a result of the intervention?
- What did you find the most helpful about the PMFM program? And the least?
- If you could change anything about the intervention, what would it be? Do you have any examples?

# 3. Important Quotes by Theme

### Theme 1: Factors Driving Positive Outcomes of the PM4M Intervention

*“The WCHW was dedicated and open. Helped my thinking.” (Participant 20)*

*“The WCHW was caring and understanding and cared for my feelings and thoughts.” (Participant 21)*

*“The WCHW scheduled appointments so it was organized.” (Participant 27)*

*“The WCHW was motivating enough and I looked forward to having the sessions.” (Participant 32)*

*“The WCHW was consistent with the sessions and physical visits.” (Participant 38)*

*“The WCHW was good with me, very patient, good explanations.” (Participant 44)*

*“Communication with the WCHW. She was very friendly and helped us to understand the intervention.” (Participant 12)*

*“She was kind and persistent.” (Participant 30)*

*“They encouraged me after I shared my problems, which helped me to stop thinking about ending my life. And I am in the process of resuming the business I gave up with. The WCHW’s call gave me hope as well.” (Participant 32)*

*“The Breathing exercise and opening up what was stressing me to the Wellbeing Health Worker.” (Participant 33).*

*“It was good to talk to someone encouraging me to do the best in my life.” (Participant 13)*

*“I changed how to handle my thoughts and emotions and how to keep going keep doing. Never to give up no matter the situation I find myself in.” (Participant 2)*

*“Exercises helped me and how to handle unhelpful thoughts, and the importance of having social support from people in the neighborhood, church and family.” (Participant 7)*

*“They were important sessions which helped me face my problems and overcome them.” (Participant 31)*

*“The lessons where helpful, especially ways to avoid suicide.” (Participant 39)*

*“Exercises were helpful. They were related to real life situations, and what I was going through.” (Participant 44)*

*“The stress relaxation techniques helped motivate me.” (Participant 45)*

*“What helped me the most was the story about Mary. I learnt that when a child makes a mistake like Mary did, the solution isn't to abandon her.” (Participant 3)*

*“Controlling suicidal thoughts I used to have. I listed my problems to solve 1 by 1.” (Participant 28)*

*“They encouraged me after I shared my problems, which helped me to stop thinking about ending my life. And I am in the process of resuming the business I gave up with. The wellbeing Health worker's call gave me hope as well.” (Participant 32)*

*“The Breathing exercise and opening up what was stressing me to the WCHW. When I am stressed I used to do the breathing exercise and I would have a calm mind and think positively afterwards.” (Participant 33)*

*“I learnt that I can control myself even when I am passing through a problem because I heard other people's problems. I saw that other people are going through difficult situations than me, so this encouraged me.” (Participant 34)*

*“I knew how to manage stress after everyone talked about their problems and we were then taught the breathing exercise.” (Participant 35)*

*“I felt encouraged with the group session.” (Participant 36)*

*“I managed to concentrate towards my children after I was taught about Mary's story.” (Participant 39)*

*“The sessions were all helpful, they should be extended to others.” (Participant 39)*

*“It has helped a lot. Every session was a reason and a lesson for me, for example, on how to relate with my kids.” (Participant 6)*

*“I started seeing a change in how I have been feeling towards unhelpful thoughts. Having a lot of thoughts would stress me a lot, not until I did a breathing exercise did I feel better.” (Participant 23)*

*“The stop light technique helped me, I do not react right there and then when something happens. And also the circle of control helped me, I am practicing this in my daily affairs.” (Participant 33)*

*“Taught on how to venture into business. I am able to provide for my family. I have no thoughts of dating someone for monetary benefits only.” (Participant 45)*

*“As a woman you are not just supposed to sit, you need to start doing something. The way I am supposed to raise my children if we are going through problems, which can affect my thoughts.” (Participant 48)*

*“Improved how to overcome unhelpful thoughts. I’ve been able to avoid bad thoughts like harming myself, blaming myself or feeling like I’m unhelpful.” (Participant 50).*

*“There is a change. I used to get involved in my parent's misunderstanding, but after learning about the circles of control, I now know things I can get involved in and what not to.” (Participant 4)*

*“Before, I didn't know how to manage my problems, but now I do. I go out to look for work to raise money for business.” (Participant 8)*

*“Positive rewarding has really helped. I used to take out my frustrations on the child and now I don't.” (Participant 24)*

*“With time they started understanding, especially by the time we were getting to session six or seven, some of them would even call to say when can you have the next session?” (WCHW 3)*

*“I think the most helpful session was the triangle, getting them to understand that everything starts from their mind. I would have some that would say, my husband has cheated in the past. So I think he’s cheating again. Then when I asked her why do you think he's cheating, have you ever seen anything that suggests that he's cheating? She says no. But why do you feel like that? She says, because he's cheated in the past. And he wasn't answering me then. But I was like, you know, he works in a factory. What if he had left his phone or he wasn't allowed to use his phone? So everything is about your feelings, your thoughts, and then your behavior. So it's a cycle that you're creating in your life. So even for you to see challenges. I think you need to start changing how you've been viewing things and how you're seeing things. Then she thought it's really helpful. So I think the important triangle really helped me for my participants, getting to explain to them how things function.” (WCHW 3)*

*“I think for me, the sessions that were most helpful, I think the session called the CBT Triangle. You see that actually, participants become aware that the way they feel the way they think and their behavior, it's something that is interconnected. It cannot be separated. So as the participants were able to identify that okay, yeah, that time when I was angry, well, I had these thoughts. And actually, I was so aggressive with my children. So they were able to actually pin the dots that okay, the way I feel my thoughts, and my behavior is actually connected and if my behavior is positive, then meaning that if maybe I'm angry, then I probably take a walk or go, maybe chat with friends or do something that I enjoy. Even my mood will change, because these things are connected. So that one was useful as well. As the decision for unhelpful thoughts. Because participants will actually be able to identify that these unhelpful thoughts are just thoughts. And not only that these unhelpful thoughts can actually be replaced by helpful thoughts. And those might my talks had to be connected to where we're looking at problem solving because now you just have to start looking at ways of solving this problem, or these unhelpful thoughts that you have. So I think that one was also helpful then lastly, the cycles of control as well. So I think the variable to realize that if I concentrate on things that I have control over, meaning, I will stress less.” (WCHW 2)*

*“I also noticed that the physical visits that we were having actually had more impact, because you would even notice, if you're in person, yes, the concentration would be there but you can tell because you'd have to maybe keep on calling out this participant just to make sure that they're with you and making sure that they are they're understanding what you're talking about. But for the physical session, you will see that the participant is actually fully attentive and you're moving together, they're able to understand. So I think for the phone interviews, I would say maybe there are times when participants were not so much as 100% committed to the activity that we're doing but for the record sessions, you could tell and you could see that they were paying attention 100%.” (WCHW 2)*

*“The other time when I was doing the session with this client, the husband was inside and we were outside with her. We were having the phone. They were today, sessions. So after I was done, then I went back home the other day, I was calling to see if I can make an appointment with her. Unfortunately, it's the husband that pick up the phone. And I introduced myself to him then he was like, wow, oh, it's you. In fact, I'm really happy that you're having these lessons. These lessons are really good, and they're really helpful. So yeah, I'll tell her in fact, when I go home, I'll tell her to say maybe you make an appointment with her so that you meet. So that was a plus to me. I appreciate it to say the man also is interested. And it's not only him in the other one also complimented on the same appreciating the innocence.” (WCHW 1)*

*“The manual was very helpful to me personally. And also the workbook was really helpful, I think to the clients, the participants, at least some of them were able to express themselves. Through writing, I realized that some participants were more open when they were writing Yes. So I think that's very much helpful.” (WCHW 4)*

*“'I’d say the manual. It was very good guidance. It gave us a guideline so we would know what we're talking about when the client is about to go offline and would bring them back to the session. And also the workbooks that we give them where they have to write their goals and everything. I'd say that was very helpful.” (WCHW 3)*

*“The introduction parts and agenda, how I should open up and explain. I think those were helpful, especially the points where you tell us what to say, how we should say. We should ask them this. I think that was very helpful, gave us good guidance. We knew how to steer the conversation and where not to go and how to guide ourselves in that was helpful for me.” (WCHW 3)*

*“The fidelity form was most helpful at the beginning because I was getting feedback and trying to pace myself and seeing where I was having gaps and needed improvement. But the manual has been helpful throughout, because it was a pointer in the way you're supposed to probe, maybe you chose if there's something that you missed, it shows you the key tips on what you are expected to maybe ask for or maybe in the special module expected to look for, or even as you're talking to the client.” (WCHW 2)*

*“The manual was very helpful to know how long was supposed to do sessions. I would know when we're going overboard or when we're probing someone who and have been on this subject for too long. We're supposed to move if maybe something says 15 minutes. We have been on it for 20 minutes. I think that was very good guidance for us. Thank you very much.” (WCHW 3)*

*“I think, first of all, putting the group session as part of the program was a very good idea because it helped the participants see that they're not the only ones who have a problem, so it was driving to the fact that people have problems, but it just comes to the fact of them knowing how to manage these problems. So it made them see that even others in that same group that we had the session in, they also had maybe similar problems or maybe different problems, but it was able to open up the fact that problems are there and there might be different kinds of problems, but people do have problems in short and I think it helped them open up as we went through session 2, session 3 and these other sessions. So I think it was helpful and for session 1, which is a group session, it was very helpful and it helped to push the objectives of the program because people were able to open up because they realize that actually I'm not the one who has this problem or this other person also has this problem, so it helped for them to open up and to be able to be receptive to some of the strategies that we talked about and to try to use them to manage their problems.” (WCHW 2)*

*“I had a few participants that failed to come in the group. They were a bit uncomfortable with people identifying and everyone hearing their problems. So the way we had 2 group sessions and 8 individual sessions, I got a lot of people to speak openly in the individual sessions, unlike the group ones.” (WCHW 3)*

*“I'm really really glad we had that training because the roleplays really helped. We got to speak with our relatives and I just imagined I was even telling my colleagues the other time after I think we got to session 2 or 3, I was like, imagine if we just got the manual and started talking to the people, would have been all over the place. So the roleplay and the training that we did, it really helped, especially with the screenings that they made us go through. So we’d prepare ourselves and just practice before going. It really helped.” (WCHW 3)*

*“The trainings were intense. They were really, really helpful. Yes, so I think if I can say the way I was before I started the intervention or, and before I went through the training, I've really seen a difference. They were packaged in such a way that, even the obstacles that I was encountering, like, during the sessions, at least I was able to handle them because of the trainings. Okay, they were really helpful, very much.” (WCHW 4)*

*“I have learnt a lot and most of it I have been able to apply it actually on myself and people around me, I would say close family, because there are certain things that are actually good to realize that, for example, if you look at the triangle, I go to realize that actually my behavior is mostly affected by my thoughts and my feelings. For those things were there but I actually got to realize it when doing the sessions. I was able to relate, but there were times when I was feeling like this and probably my behavior would be like this. And then when you go to the end of the session, if you talk of having positive behavior of which I actually did practice on myself and I saw that actually had a positive impact. Then, I also agree with the circles of control, which are very important and have actually helped me through a number of difficult times where I try by all means not to concentrate on the outer part of what other people probably might say to me or what they might think because I don't have any control over that, but then I just try to concentrate on what I have control over. So that I think has helped me as well, as well as the stoplight technique. It also helped because now I don't jump into conclusions, but I actually take time to actually think and evaluate the situation, probably what I'm thinking would have caused the situation for it to be like that is actually not the case if I relax and I'm able to evaluate the situation. I've actually been able to relate to I'd say most of the techniques and the sessions, the session lessons that we've had and I’ve actually given examples to even people that are around me, to try them because I've seen them work, so the training has really been helpful and impactful.” (WCHW 2)*

*“I found the circles of control to be more helpful for me, it has really worked for me because if I look a while back when I didn't know about the circles of control, like how to deal with, maybe let's say, I would maybe constantly think about things that are outside of my control, but when I learned about the circles of control, I feel more positive, I know that not all things, things that are not in my control, I'm not supposed to worry about them. I'm supposed to concentrate on things that are out of my control. Those are things that are out of my control. I’m not supposed to worry about them. I'm supposed to concentrate on things that are within my control. So this has really helped me and also in terms of how I can interact and relate with people of various backgrounds. This is something that I've noticed, I've noticed a certain change, a certain shift and perspective that I have towards people on how I can relate with them, on how I can interact with them, and also the other thing that I can say is also about assertive communication. I'm one person, I was one person maybe if I have an opinion and everything, I will just be there. I wouldn't even do anything about it, but I think I've seen a change in the way that I communicate with others. More assertive and also, okay it's a lot of things. Also, my social support. I think I’m one person who used to neglect this one aspect to say okay just maybe let me just tell a few people that are my social support, but now my social network has increased, especially at church. Yes, because I knew that this is one aspect that is very cardinal in my life. So I really appreciate this program. It has really helped me and taught me a lot of things that I didn’t know. Okay.” (WCHW 4)*

*“For me, I think Dr. Thandiwe was very helpful in where I would have trouble or where the client was not responding in times when I would feel like giving up maybe I call her and I tell her no she's saying this, she says she's not ready anymore, she's feeling like this, she feels like she wants to give up. So she would tell me, no, just go back in and tell her and then after she's told you that tomorrow morning, call her. She was very very helpful. She would guide me through it and she would check up and see, did you speak to that one? She would follow up after and guide me, so she was very very helpful. I’m very pleased.” (WCHW 3)*

*“I had the same supervisor as WCHW 5. I would say she was really helpful. Yes, when I don't, maybe when I have an issue, she would follow up, we would have calls maybe within more than 30 minutes she's just explaining to me on how I'm supposed to do and how I'm supposed to go about the whole thing and yeah she used to check up on me, find out what I'm doing, how, like how the whole things, the whole like program is going and everything, so yeah that on itself was really a motivating factor for me to push and continue with the sessions. So she was helpful.” (WCHW 4)*

### Theme 2: Understanding the Positive Impacts of PM4M

*“The time I had session 4 it helped me greatly because I had marital problems, but after attending the session it helped to keep going and not give up.” (Participant 6)*

*“Exercises helped me and how to handle unhelpful thoughts, and the importance of having social support from people in the neighbourhood, church and family.” (Participant 7)*

*“What helped most was the relaxation exercises that I was taught,. And it has helped me have a positive thinking from a negative thinking. I was able to manage my stress.” (Participant 12)*

*“My WCHW was caring and understanding and cared for my feelings and thoughts.” (Participant 21)*

*“The WCHW was motivating enough and I looked forward to having the sessions.” (Participant 32)*

*“What helped me the most was the story about Mary. I learnt that when a child makes a mistake like Mary did, the solution isn't to abandon her.” (Participant 3)*

*“Communication with the WCHW. She was very friendly and helped us to understand the intervention.” (Participant 12)*

*“They encouraged me after I shared my problems, which helped me to stop thinking about ending my life. And I am in the process of resuming the business I gave up with. The WCHW’s call gave me hope as well.” (Participant 32)*

*“When I am stressed I used to do the breathing exercise and I would have a calm mind and think positively afterwards.” (Participant 33)*

*“I learnt that I can control myself even when I am passing through a problem because I heard other people's problems. I saw that other people are going through difficult situations than me, so this encouraged me.” (Participant 34)*

*“It taught me how to manage my stress as a single mother.” (Participant 36)*

*“When I am upset I am not supposed to project my anger towards my children, I am supposed to show them love.” (Participant 38)*

*“I managed to concentrate towards my children after I was taught about Mary's story.” (Participant 39)*

*“How to take care of a child. Feeding them, keeping them clean, ensuring they take naps. Not always falling for the child's wants when they cry or display bad behaviours.” (Participant 45)*

*“Stopped paying attention to people who tease or speak down on her and she keeps to herself.” (Participant 48)*

*“I am able to regulate my emotions and control more of my thoughts than before.” (Participant 50)*

*“Problem sharing, she never used to share problems with friends and it helped her feel lighter with less burdens.” (Participant 53)*

*“It has helped me greatly in the fact that I need to socialize with my neighbors, community and friends.” (Participant 1)*

*“It has helped me in my problems that I had, and the problems ahead of me. And it has helped me know how I am supposed to live with people in my community.” (Participant 2)*

*“It has helped a lot,every session was a reason and a lesson for me, for example, on how to relate with my kids.” (Participant 7)*

*“It helped me how to challenge my thoughts on how to take care of my child.” (Participant 10)*

*“It helped positively with regards to children. When upset, the anger or stress used to extend to children. I stopped shouting at children when stressed.” (Participant 15)*

*“I used to be suicidal and had problems now I control my thoughts.” (Participant 20)*

*“I used to wake up moody and pick fights with people, now I communicate well.” (Participant 24)*

*“I used to be depressed and be harsh on kids, now I am good to them.” (Participant 25)*

*“I learnt how to handle problems and how to solve them. And how I can think positively about what I might find myself going through. Having a bad thought does not make it real.” (Participant 32)*

*“I am able to share certain things I used to keep to myself before even when it requires me to share. As a result I feel relieved.” (Participant 37)*

*“I used to panic when I found myself in a problem, but now I am able to manage problems well. I distract and keep myself busy when I am having negative thoughts. And when I start something, I am not supposed to stop not until it’s fulfilled.” (Participant 35)*

*“I do not overthink things now, I communicate to my family what I’m going through, which was not the case before.” (Participant 38)*

*“I am different from how I used to talk to people. I think before I speak.” (Participant 42)*

*“It helped a lot because I am now able to socialize with people in a good way. Before I would get upset and aggressive towards anyone who said bad things about me.” (Participant 44)*

*“There's a great change on how I treat my children. I used to beat them, but now I know how to control myself.” (Participant 1)*

*“There's a change. For example, before the intervention, I used to think of suicide everytime I encountered a stressful event. Now, I have changed how I think and how to solve my problems. Even my family members are surprised on how different I handle my problems now.” (Participant 2)*

*“I never used to exercise, but now I do.” (Participant 3)*

*“I managed to change my thoughts and found a job.” (Participant 13)*

*“I have a garden and sell vegetables from the same garden.” (Participant 15)*

*“I used to be isolated, now I've made friends.” (Participant 23)*

*“I improved healthwise, I even gained weight and stress less.” (Participant 29)*

*“I have started a business and I will not stop because I need to keep on going and keep on doing. Before I used to stop when I would face difficulties in my business.” (Participant 33)*

*“I stopped the job I was doing and have started a business which is sustaining me rather than that stressful job where I was not getting paid a lot of money. I can now save money, and I keep on going.” (Participant 34)*

*“I do not react easily when my husband is talking to me.” (Participant 35)*

*“I am not embarrassed about selling things as a source of income anymore. I have gained confidence.” (Participant 42)*

*“I have learnt that I do not need to be like Mary's parents and abandon my children when they get pregnant out of wedlock.” (Participant 3)*

*“I learnt how not to stay awake up to awkward hours, after learning session 8. I now have good sleep through the strategies.” (Participant 4)*

*“It was good to talk to someone encouraging me to do my best in life.” (Participant 13)*

*“I have happiness. I think well before I speak. In my house, I now have peace. My children are happy seeing a new nice mother in me.” (Participant 16)*

*“My clients came to appreciate session seven, where we talked about the circle of control because most of them talked about how they didn't know how to control their situation. So after learning about the circle of control, they think they now know what the focus of the thoughts should be if it's outside the scope of control. They now know they shouldn't even bother to think about that same thing. And also session five, which is how to challenge their thoughts. It's just a thought they now know how to challenge their thoughts and they now know that thoughts can just be thought sometimes and not really something to do. They came to appreciate that because now they know how to handle their problems when a certain situation comes.” (WCHW 5)*

*“I think, for me, the session which was the most helpful, even for my clients, is session five, unhelpful thoughts. I realized most of the clients, the problems that they were facing, it's a result of the way they were processing things. The unhealthy thoughts that they were allowing. That's what was contributing back to their problems. So I think they also reported that because of the same lesson, they are able to process their thoughts well, they are able to know to distinguish between which thoughts are helping them and which thoughts are not helping them.” (WCHW 4)*

### Theme 3: Intervention Delivery and Format. Key Points for Engagement Success.

*“We did not finish up to session 10, because of little time. The WCHW would come and would not find me home.” (Participant 9)*

*“I would encourage that you take it further to other moms who did not receive the intervention.” (Participant 6)*

*“Providing transport.” (Participant 16).*

*“Sessions 1 to 10 should be done as a group, and a specific venue should be found.” (Participant 36)*

*“The program should be extended to a lot of women because it's helpful. The sites targeted for these women should be under 5 clinics.” (Participant 50)*

*“Having a show on radio in order to have a larger audience because the topics are good and encouraging and would benefit a lot more people on a platform like radio unlike one on one sessions. Even having group sessions, unlike phone sessions.” (Participant 51)*

*“To continue and also make us teachers in the community.” (Participant 21)*

*“More people should be included in the program. I once explained to someone who asked me of this program, and they seemed interested in joining it.” (Participant 35)*

*“The other challenge we had is the group of people were dealing with. Most of them didn't understand the importance of what we were trying to bring across. So they were more used to receiving monetary gifts or maybe physical gifts. But with time, especially when the session was starting, they didn't understand them. A lot were hiding from us and skeptical.” (WCHW3)*

*“I think the challenge was just getting them to understand that their mental well being was vital and they couldn't be physically able to look after themselves without taking care of the mental state first.” (WCHW 3)*

*“I think there was an expectation of money of some sort. And I think for the few that we lost along the way, the inconsistencies that we had, it will draw us back to the monetary gain that these people were actually looking at.” (WCHW 2)*

*“The sessions were really helpful for some of the respondents, but for others, they didn't see the importance of the sessions, because they were expecting something. Do they expect money? Like for each session, you're expecting maybe to be given something? Because I feel that it's like they're used to being given things when they're interviewed? So like, this was something new to them.” (WCHW 4)*

*“I think session eight because most of them didn't have problems with alcohol abuse. They didn't have problem with sleeping pattern. They didn't have a problem with nutrition and so they said on that one I it's more like I just did that session for formalities sake because most of them didn't have those problems, any of those problems.” (WCHW 5)*

*“The least helpful was session 10 because I think it was more of a goodbye as long as they've gathered all the knowledge, but session 10 to me seemed like was all the things that they really needed to know, like how to keep themselves healthy, how to avoid unwanted thoughts, how to avoid unhelpful thoughts and things like that even how to know positive rewarding, all those things were covered. So session 10, for me, was the least helpful.” (WCHW 3)*

*“When it comes to the least helpful sessions, I think session 9 and session 10. If maybe there was a way to incorporate these two. What we covered in session nine is a bit similar to what's in session 10. So like some of the things are repeated.” (WCHW 4)*

*“For me one challenge that I found was communication. I'm thinking maybe if it was very difficult to communicate with the clients, it's because these are people. When it rains, the houses are flooded, so they will move and then it's very difficult to find where they stay, and most of the housings don't have a house number, so you have to move in the community. So it's small, makeshift homes. So it was very difficult to get to the client. Some of them we didn't even get to finish, some of them even stopped along the way. It was mostly communication. Some didn't have phones. So I'm thinking maybe if they could have communication, maybe phones, because a lot of them didn't have phones.” (WCHW 3)*

*“I also noticed that the physical visits that we were having actually had more impact, because you would even notice, if you're in person, yes, the concentration would be there but you can tell because you'd have to maybe keep on calling out this participant just to make sure that they're with you and making sure that they are understanding what you're talking about. But for the physical session, you will see that the participant is actually fully attentive and you're moving together, they're able to understand. So I think for the phone interviews, I would say maybe there are times when participants were not so much as 100% committed to the activity that we're doing but for the in person sessions, you could tell and you could see that they were paying attention 100%.” (WCHW 2)*

*“I also agree. I think their physical sessions were more helpful because even during the relaxation technique on the phone it was very difficult because sometimes I think the client would even move out. You're just talking to yourself, but with the physical. You're able to see it okay. The client is also with me, and she's also doing the exercise together with me. Yes, so I do agree with my colleagues.” (WCHW 5)*

*“For me the obstacles that I encountered is concerning participants who resided in dangerous areas. It was really hard for me to communicate with them. Especially if they don't have a phone so it means I'm forced to go to their places where they stay. That was a bit dangerous. Yes, there are some areas which are dangerous. One of my participants was staying at a bar.” (WCHW 4)*

*“But in addition I think maybe, as I just said, maybe having more physical sessions, maybe even 80% or 90% physical sessions as compared to phone calls. And as well as probably trying to have a neutral place where these people can can where we can be having this session. Most of the times we were doing the sessions in their homes. So you will find that they have marital problems. So you will find that probably the husband is even around when you're having this session and it compromises. The information you're going to get from this participant or they're not going to be open because for one I had a participant who I could tell she was not opening up a number of times because the husband was in the house and there's a time I had a physical session with her. And we had it from the mother's house. So she was a bit more open this time around as compared to the times that the sessions were actually happening. Maybe in her house or maybe just outside the house and her husband is around.” (WCHW 2)*

*“I noticed that my participants preferred conversations, because one time I was doing a physical session with my participant. And then we had it from outside their place, but people were passing and everything and after I was done when they spotted me then people are like, why are you doing consulting sessions? Is it because they associated with HIV counseling and being positive? That's why this person is coming here looking for you and everything. So I think just from that time, I noticed that participants seemed uninterested, and we couldn't even finish the sessions. Each time I go there physically, she'll just be like, Oh, no, I'm busy. So I think the physical sessions and the phone sessions also depends on the preferences of the participants or a neutral place where the sessions can be conducted so that not everyone knows about it, because here in our, let's say, the African settings, it's not a common thing. For someone to go for counseling, and it's like a new theme, because people don't really pay attention to their mental health or the things that they're going through. So maybe they could find a neutral place where they can be emitted from.” (WCHW 4)*

*“Some people don't take mental health that seriously. I think that's why we've had a lot of suicide. Suicide situations in the past because they're not really serious with mental health. So maybe if we had a neutral place where they could come in at the clinic, or at least go somewhere. And also the other thing is, maybe if I could change something about the intervention maybe I would say next time, maybe we get those women who are willing, like they volunteer on their own to say, look, I want to be part of this program. I think maybe we could have something where women come on their own to say, look, I'm having difficulties, depression, stress and all that and I really want to be part of this program.” (WCHW 5)*

*“The fidelity form was the least helpful, though it was also helpful but it can't compare to what the manual provided.” (WCHW 3)*

*“Although I think for the workbook, maybe we needed to take a little more time on trying to explain how it works, because I have some participants that probably couldn't read or maybe couldn't read.I think one or two. So it was kind of maybe a bit of a challenge on their part.” (WCHW 2)*

*“Some of them could not relate with some parts like activities like jogging or going for a drive. I think those are things that are good for them. I think they'll think you're pushing them too far. They're telling you that they've slept hungry and you're telling them to go jogging or drive just to clear their head. So I think some of those things were not culturally adapted. The things that they liked are things like gardening, I think those are related. Well, those were okay. Things like gardening or just leaving the house to take a walk and reflect to just get a different feel so that they covered the stress. And also the fruits. You're mentioning that I should include fruits in my diet. So I think some of those were a bit different from their culture or the setup that they were used to. So we just have one meal and I've been feeling so depressed everyday. I'm even thinking of maybe finding poison and just dying because we don't have anything to eat. So like telling the good fruit, that was a bit touchy for them. So I think some of those I wouldn't go, I didn't go that deep into advising them to do that. I wouldn't mention them.” (WCHW 3)*

*“I can relate to what P3 is saying. It's like I'm telling someone who was struggling financially to be taking at least five fruits in a day. When they don't have every meal, when they're just maybe eating vegetables and everything I'm telling them to do like a balanced diet and everything, they were just like, okay, maybe when I have some of the things. Depending on the participants' situation, I think it was not relevant to them, some of them. So I was just sensitive when I was even just giving all the information to them.” (WCHW 4)*

*“I also agree that there were some parts in the manual where we had to maybe just try and not do away with them, but try and adjust to the setting and maybe the person that we're dealing with at that particular time. And I think the other thing that I would say is, I think like P4 said, I think it's in far the beginning where, like, in our setting like even in the areas that we were maybe the areas that were being targeted, a lot of people are not so used to having counseling or receiving any kind of therapeutic intervention in terms of maybe mental health or any form of counseling because for them, they understand that counseling is mostly attached to HIV and AIDS, and maybe HIV and AIDS counseling and testing. And so, I think for the first part, I could see that in some people we were struggling and especially even the part of where we have the techniques, the stress management techniques, so people are not used to this. And so it's something that I would say is quite new to them. It's something that maybe they've heard of but maybe they've never really done it and so it was something that was like starting something new if I could say so it's something that is not so culturally innate in our country.” (WCHW 2)*

*“I also noticed that in session 3, we ask them about problem solving. So to list the problems, see what they are able to do when you're asking them to do that. They start talking about businesses, like I don't think they understood that we wanted to make them think outside the box. Think of things that they can do. I think they thought we were trying to tell them no, we want to empower you. So I think some of those that didn't understand, when they started understanding, I think between session 4 when you ask them what can you do, what do you love to do? What can you work with around you? It's those that started falling out because they saw that it was about their mental health, not about funding or environment. So I think those that noticed that it was not about empowerment, they started pulling back from us just after session 3.” (WCHW 3)*

*“If we can’t afford breakfast or lunch, your fruit we are struggling to find. So you are there just explaining the good part of having a fruit a day and you encourage them. So there were some challenges. And in the part of session 3 where you're teaching someone to problem solve, maybe we can start having them ask someone what they can do. And then you'd see from there, the way they would come out with the hope to say maybe they'll help us. So it was a little bit challenging.” (WCHW 1)*

*“I think the other challenge is that Zambian women are not familiar with saying okay, I can be taking antidepressants, the medications for mental health related illnesses. I think those are not things that they are familiar with. And then the other challenge that I found was that this respondent I reported to say, okay, she's having problems and she needs to be attended to urgently. You should come at this facility and everything. And if they associate the same facility with people who are maybe mad or something when they hear that particular facility, what comes to their mind is maybe they're not okay. So when they caught that, she said, I'm not available. Yes, that's what she told me that's what you put it because they have that perceived perception to say when someone is going to those facilities, mental health facilities, maybe they have diagnosed them to be mad or insane.” (WCHW 4)*

*“I think the way the arrangement of the sessions was, it was just okay. It needed maybe let's say more group sessions because I feel in group sessions that's where individuals or participants express themselves more as compared to maybe when I’m just with them like just the 2 of us. Yeah, that's the distinction I made with the group sessions that I had to say. Most people tend to open up more even when they are in a group, some of them.” (WCHW 4)*

*“I had a few participants that failed to come in the group. They were a bit uncomfortable with people identifying and everyone hearing their problems. So the way we had 2 group sessions and 8 individual sessions, I got a lot of people to speak openly in the individual sessions, unlike the group ones.” (WCHW 3)*

*“I think just like WCHW 1 mentioned, some sessions were a bit too long. Some were short like session 4, like keep going keep doing, and I think session 8 was a bit too short.” (WCHW 3)*

*“But for the other ones, like session 8, it turned out that 1 hour was a bit much. So you would find maybe by the time we're looking at the 45 to 1 hour, the client is not responding.” (WCHW 3)*

*“I do agree with WCHW 3 on that one. I also feel session 8 was too long, considering the fact that there wasn’t anything to talk about except for sleeping patterns and deficiency.” (WCHW 5)*

*“So just saying something on the same session, my 2 colleagues have mentioned on session 8. Yes, it was too long only if the respondent maybe had no problem with their diet and maybe had no issues to do with their sleeping pattern.” (WCHW 1)*

*“You are just trying to encourage the person to take care. Yeah, it was more like you are just encouraging the person if you find that the person had no problem.” (WCHW 1)*

*“So I think I've got two additions that I think would probably improve the program. The first one I think I had mentioned it as well, where we're having maybe a neutral place, not their homes, where we can have the sessions from so that, maybe they are, for lack of a better term, more open without any disturbances.” (WCHW 2)*

*“To me the supervision was 50/50. Why did I say 50/50? She helped me, yes, a lot and I really appreciate it. But there are times she would go quiet. Maybe that quietness meant I was doing well. And maybe I needed less attention, like maybe she thought I was doing okay.” (WCHW 1)*

*“I think what can be improved when it comes to the supervision is implementing, maybe when an issue is raised, let's say when a WCHW raises an issue to say this is the problem that they are facing with their clients, so just implementing it there and then. I noticed I would raise an issue to say this is what this person is going through. Maybe they want to end their lives, or maybe they are threatening to end their lives and everything or maybe the responses that they are giving. So, maybe if I raised that issue to my supervisor, maybe they would follow up, just after some time. Yeah, so I was thinking if an issue is raised, to say this person or this respondent, this is what they are trying to do. I think they should be implementing it right there and then so that that person or those people can be helped.” (WCHW 4)*

*“As we go there in the field, we are the ones that are on the ground and we interact with these people, we see and hear from them. So if something is said, maybe reported to the supervisor, the quickest response should be there, yes, so that at least things are even easier for the person that is on the ground.” (WCHW 1)*

*“Yes, I agree with WCHW 1 and WCHW 4 on the supervision part, where I think next time they should move quicker when someone raises an issue. You know, for a plan towards committing suicide, and like maybe for them to respond to that, especially after 2 weeks, or 1 week, I don't know. And also the other thing was, maybe if we could just like maybe every day, the supervisor would check up on the WCHW. Like how were the sessions today and how did it go? Did you have any challenges? I think that would be a motivating factor as well.” (WCHW 5)*

*“Okay, maybe the supervisors next time if they would coordinate or maybe like work hand in hand. Like maybe when managing certain cases, I don't know how it was done. The coordination was not that much. Yeah, so maybe they could work hand in hand, coordinate certain cases and everything. That would really be helpful.” (WCHW 4)*

*“Another challenging part, maybe with the management here, the organization here. So the thing is maybe not understanding one or two things, then you ask for clarifications. Yes, there you are told, but again, some days pass and again you don't see it being implemented. Again, you follow it up like that. So mostly I can say on the challenge part yes, things were being delivered. Yes, in terms of mandatory forms for the ones we use for transport. Yeah, but maybe you'd wait for some days, just waiting on the money for you to go in the field. So, yes, they were being delivered after some time, but that period of waiting, because there are those procedures of going to the accountant, yes. But at least if they are put in order so that they don't inconvenience us as well.” (WCHW 1)*

*“I agree with WCHW 1. I believe resources used to come, but they used to take time. For example, we would use talk time like on a regular basis, so it's something that should probably be flowing and you don't have to remind them that we need talk time. Or it's something that should have been a consistent kind of flow because we were constantly using talk time whether we were doing physical visits, whether we were doing phone sessions, so just as WCHW 1 said, we would request for talk time, but it would take some time to come to probably you would even have to maybe reschedule your sessions because you don't have the necessary resource at that particular time. Yeah. So I think resources were coming, but they were not really coming as consistently out as or at the right time as they needed to come.” (WCHW 2)*

*“I gave an example of talk time. You would maybe say, I need talk time on a Monday, but the talk time would only come through maybe after 3 days. My thought would be, for example, if we were being given talk time maybe every 2 weeks, meaning that in the second week, maybe a follow up should have been taking place, maybe on a Friday, to find out when it is and find out if we needed more talk time, so the following week when we’re supposed to have probably additional talk time, the talk time would be available so that way it is flowing smoothly with the needed talk time.” (WCHW 2)*

### Theme 4. Recommendations for Scaling PM4M.

*“What I encourage is that I would be empowered with business capital after the intervention.” (Participant 1)*

*“I would change that you bring empowerment programmes next time.” (Participant 3)*

*“If resources allow, I would change that you start some employment program.” (Participant 8)*

*“Continue helping but also include financial empowerment help.” (Participant 20)*

*“Helping those people in need financially with capital. To empower people . We may work but we don’t get enough help so adding a little empowerment or capital or clothes.” (Participant 24)*

*“At least provide something as you make calls. Financial aid.” (Participant 28)*

*“At least empower us or offer something. I didn't receive anything since inception up to date.” (Participant 29)*

*“Include our partners in the programs. Even through the phone. Include the men because even the funeral next door is a male suicide. Men are also very stressed.” (Participant 21)*

*“For us to continue feeling good, they should be providing capital for us. So that we start businesses.” (Participant 32)*

*“Empowering the women with capital, entrepreneurship skills and finding for them what can keep them busy so that they are able to take care of their children nicely and not go into alcohol abuse. And so that their children do not grow up to be junkies.” (Participant 34)*

*“Include capital to help with my small business.” (Participant 46)*

*“Helping us start up a business. For example, giving the women in this program a loan to start up something. Because staying without doing anything is what brings about most bad thoughts.” (Participant 49)*

*“Most of my clients were expecting something in return, either after the sessions were done, or during the sessions, and most of them I think the reason why they were even reluctant to have the sessions was because they kept on saying, I've told you that the reason why I'm having so many thoughts is because I don't have anything to do. I’m not having unhelpful thoughts. I'm going into depression because I don't have any business. So I think they were expecting some more empowerment after the intervention. Or during the intervention.” (WCHW 5)*

*“I think I would add the partners because it seems that most of the challenges they go through, the partners are a contributing factor. If the male partners could be added or maybe there was a program for their partner for the well being of the child, they can work together because you find some of their husbands abused alcohol and were not using decent language. So if the mother is doing that and the husband is doing the opposite, I think the children might get confused. If there was a male intervention, they could work together for the wellbeing of the child.” (WCHW 3)*

*“So you will find that they have marital problems. So you will find that probably the husband is even around when you're having this session and it compromises. The information you're going to get from this participant or they're not going to be open because for one I had a participant who I could tell she was not opening up a number of times because the husband was in the house and there's a time I had a physical session with her. And we had it from the mother's house. So she was a bit more open this time around as compared to the times that the sessions were actually happening. Maybe in her house or maybe just outside the house and her husband is around.” (WCHW 2)*

*“So if men would be included in the program as well, that would be very beneficial and helpful.” (WCHW 4)*

*“And I think if I was to change anything about the intervention, I think some of the times the main problems come as a result of not being financially independent. So they are too dependent on their husbands to provide them with literally everything that they need. So maybe if there was a way to just offer an empowerment program or just to teach them maybe on how they can venture into things that can make them become financially independent so that they don't completely rely on their husbands to do just everything for them. So some of the problems they are facing, mental problems, will even reduce if they are financially independent.” (WCHW 4)*

*“I think in a situation where the client mentions that the problem comes from their spouse or the person that they live with, I think I would add in future, I would add that person just for them to know how they're going to work together and try to live in a more decent environment. It seemed like we were trying to work on one person when everyone around them was falling apart in creating commotion.” (WCHW 3)*

*“From my observation, most of the problems or the stress or any kind of depression, the root cause of this for most of the participants that I had was financial. So maybe if there can be a component to add on to the program where they can be helped further to start a small business in order to maybe completely improve on their wellbeing because if we are going to talk to them quite alright they are going to find ways but at the end of it if the root cause is the financial stressor, as long as that problem is not worked on or is not improved, meaning that at some point it can keep on coming. So maybe also factoring in that part of trying to help them not only mentally with mental health with the sessions, but also maybe an empowerment of some sort.” (WCHW 2)*

*“I do agree with what my colleagues have said. If maybe we could add a small component, even maybe just teaching them a second skill, whereby literally they source for their own business capital, and they start doing the skill that way, because even during the endline, most of my clients or most of the participants on that question where it was asking them if they would like to change anything about the intervention, most of them were mentioning about the same thing where if only maybe you could give us even capital to start a business or even teach us some skills and then they can source for own money to start a business, that would be really helpful.” (WCHW 5)*

*“Also, like WCHW 3 mentioned, if next time we include their spouses or if the participant’s problem is maybe family dispute, maybe we add that component of how to deal with such a thing for their mental wellbeing.” (WCHW 5)*
